# Extracorporeal treatment for carbamazepine poisoning: Systematic review and recommendations from the EXTRIP workgroup

**DOI:** 10.3109/15563650.2014.973572

**Published:** 2014-10-30

**Authors:** Marc Ghannoum, Christopher Yates, Tais F. Galvao, Kevin M. Sowinski, Thi Hai Vân Vo, Andrew Coogan, Sophie Gosselin, Valery Lavergne, Thomas D. Nolin, Robert S. Hoffman, on behalf of the EXTRIP workgroup

**Affiliations:** ^a^ Division of Nephrology, Verdun Hospital, University of Montreal, Montreal, QC, Canada; ^b^ Emergency Department and Clinical Toxicology Unit, Hospital Universitari Son Espases, Palma de Mallorca, Spain; ^c^ Getulio Vargas University Hospital, Federal University of Amazonas, Manaus, AM, Brazil; ^d^ Purdue University, College of Pharmacy, Department of Pharmacy Practice, Indianapolis and West Lafayette, IN; ^e^ Department of Pharmacy and Therapeutics, Chinle Comprehensive Health Care Facility, Indian Health Services, Chinle, AZ, USA; ^f^ Emergency Medicine and Medical Toxicology Service, McGill University Health Centre, Montréal, QC, Canada; ^g^ Department of Biology, Hôpital Sacré-Cœur de Montréal, University of Montreal, QC, Canada; ^h^ Department of Pharmacy and Therapeutics, and Department of Medicine Renal Electrolyte Division, University of Pittsburgh Schools of Pharmacy and Medicine, Pittsburgh, PA, USA; ^i^ Departments of Medicine and Emergency Medicine New York University School of Medicine, New York, NY, USA

**Keywords:** Hemodialysis, Hemoperfusion, Overdose, Anticonvulsant

## Abstract

*Context*. The Extracorporeal Treatments in Poisoning (EXTRIP) workgroup was created to provide evidence and consensus-based recommendations on the use of extracorporeal treatments (ECTRs) in poisoning. *Objectives.* To perform a systematic review and provide clinical recommendations for ECTR in carbamazepine poisoning. *Methods*. After a systematic literature search, the subgroup extracted the data and summarized the findings following a pre-determined format. The entire workgroup voted via a two-round modified Delphi method to reach a consensus on voting statements, using a RAND/UCLA Appropriateness Method to quantify disagreement. Anonymous votes were compiled, returned, and discussed in person. A second vote determined the final recommendations. *Results*. Seventy-four articles met inclusion criteria. Articles included case reports, case series, descriptive cohorts, pharmacokinetic studies, and in-vitro studies; two poor-quality observational studies were identified, yielding a very low quality of evidence for all recommendations. Data on 173 patients, including 6 fatalities, were reviewed. The workgroup concluded that carbamazepine is moderately dialyzable and made the following recommendations: ECTR is suggested in severe carbamazepine poisoning (2D). ECTR is recommended if multiple seizures occur and are refractory to treatment (1D), or if life-threatening dysrhythmias occur (1D). ECTR is suggested if prolonged coma or respiratory depression requiring mechanical ventilation are present (2D) or if significant toxicity persists, particularly when carbamazepine concentrations rise or remain elevated, despite using multiple-dose activated charcoal (MDAC) and supportive measures (2D). ECTR should be continued until clinical improvement is apparent (1D) or the serum carbamazepine concentration is below 10 mg/L (42 the μ in μmol/L looks weird.) (2D). Intermittent hemodialysis is the preferred ECTR (1D), but both intermittent hemoperfusion (1D) or continuous renal replacement therapies (3D) are alternatives if hemodialysis is not available. MDAC therapy should be continued during ECTR (1D). *Conclusion*. Despite the low quality of the available clinical evidence and the high protein binding capacity of carbamazepine, the workgroup suggested extracorporeal removal in cases of severe carbamazepine poisoning.

## Introduction

The Extracorporeal Treatments in Poisoning (EXTRIP) workgroup comprises international experts representing diverse specialties and professional societies (Table 1) brought together to provide recommendations, based on evidence and consensus, for the use of extracorporeal treatments (ECTRs) in poisoning (www.extrip-workgroup.org). Rationale, background, objectives, complete methodology, and its first recommendations have been published.^1–6^ The following text reviews the recommendations for carbamazepine.

**Table 1.** Represented societies.

American Academy of Clinical Toxicology	European Renal Best Practice
American College of Emergency Physicians	European Society of Emergency Medicine
American College of Medical Toxicology	European Society of Intensive Care Medicine
American Society of Nephrology	French Language Society of Resuscitation
American Society of Pediatric Nephrology	German Society of Nephrology
Asia Pacific Association of Medical Toxicology	International Pediatric Nephrology Association
Australian and New Zealand Intensive Care Society	International Society of Nephrology
Australian and New Zealand Society of Nephrology	Latin American Society of Nephrology and Hypertension
Brazilian Association of Poison Control Centers and Clinical Toxicologists	National Kidney Foundation
Brazilian Society of Nephrology	Pediatric Continuous Renal Replacement Therapy
Brazilian Society of Toxicology	Pediatric Critical Care Medicine
Canadian Association of Poison Control Centres	Quebec Association of Emergency Physicians
Canadian Association of Emergency Physicians	Quebec Association of Specialists in Emergency Medicine
Canadian Society of Nephrology	Quebec Society of Nephrology
Chinese College of Emergency Physicians	Renal Association
Chinese Medical Doctor Association	Society of Critical Care Medicine
European Association of Poison Centres and Clinical Toxicologists	Spanish Clinical Toxicology Foundation

## Pharmacology

Carbamazepine has a structure similar to that of tricyclic antidepressants and is used for the treatment of bipolar disorder, neuropathic pain, hyperactivity, and seizure disorder. It inhibits the release of glutamate and similar neurotransmitters via blockage of presynaptic voltage-gated sodium channels in the central nervous system (CNS). It also blocks N-methyl D-aspartate and adenosine receptors.^7^

Carbamazepine has a molecular weight of 236 Da and is highly bound to both albumin and alpha-1-acid glycoprotein (70–80%), a percentage that does not appear to decrease significantly in overdose: in one series of 4 patients with supratherapeutic concentrations, protein binding remained at 74–82%,^8^ while it decreased to 57% in another report.^9^ Carbamazepine has a slow rate of dissolution, which results in erratic and incomplete absorption.^10,11^ It is highly lipophilic and distributes rapidly and extensively (volume of distribution ranges from 0.8 to 1.4 L/Kg).

Carbamazepine is extensively metabolized in the liver by the cytochrome P450 system. Only 1–3% of the drug is excreted unchanged in the urine. Its primary metabolite, carbamazepine-10,11-epoxide, is 50% protein-bound and shares an equal anticonvulsant and toxic effect.^8^ The nature of carbamazepine metabolism is complicated by the fact that it induces its own metabolism with chronic use; this auto-induction occurs relatively early in therapy, is dose-dependent, and explains why carbamazepine-naïve patients usually exhibit more toxic symptoms at a given exposure than those who use it therapeutically.

Carbamazepine's half-life with initial dosing is reported to be 25–65 h, which decreases to 12–17 h with repeated or continued dosing. In overdose, much longer apparent half-lives are reported,^10,12^ likely reflecting ongoing absorption, impaired elimination, or some combination of both processes.

## Overview of carbamazepine poisoning

Toxicity from carbamazepine overdose was first described in 1967^13^ and continues to be responsible for a large proportion of life-threatening cases among anticonvulsant poisonings. Data from the US Poison Control Centers documented 4149 toxic carbamazepine exposures in 2012, 14% of which had at least a moderate effect.^115^

Serum carbamazepine concentrations can be used to confirm exposure. The therapeutic concentration range is 4–12 mg/L (17–51 μmol/L) (Table 2); significant toxicity usually occurs over 40 mg/L (169 μmol/L),^14^ but also potentially at lower concentrations.^15^

**Table 2.** Carbamazepine physicochemical and toxicokinetic data.

Molecular weight	236 Daltons
Volume of distribuition	0.8–1.4 L/kg
Protein binding	75%
Oral bioavailability	80–100%
Therapeutic range	4–12 mg/L (17–51 the μ in μmol/L looks weird.)
Toxic exposure	> 20 mg/kg
Toxic blood concentrations	Adults: > 20 mg/L (85 the μ in μmol/L looks weird.)Children: > 12 mg/L (51 the μ in μmol/L looks weird.)

Neurologic symptoms including movement disorders, altered mental status, and seizures primarily characterize carbamazepine toxicity. Respiratory depression is common in severe overdose and can be complicated by concomitant aspiration. Cardiovascular effects include sinus tachycardia, hypotension, myocardial depression, and cardiac conduction disturbances; in rare cases, QRS complex prolongation, bundle branch block, Brugada-type patterns, atrioventricular block, and premature ventricular contractions are reported.^16–18^ Death has been reported due to refractory cardiovascular toxicity.^18–20^

Medication clumping and slow dissolution may occur with standard and sustained-release formulations.^21,22^ At high concentrations, carbamazepine exhibits anticholinergic proprieties, which delays gastrointestinal motility further prolonging absorption, with peak absorption sometimes documented over 100 hours post-ingestion.^12,17,23^ Severely poisoned patients may suffer clinical deterioration after initial improvement or delayed onset toxicity that may result from rebounding or persistently high serum concentrations. Hyponatremia is a frequent adverse event during treatment, but is uncommon in cases of acute poisoning. Fatalities are extremely unusual,^24^ but in one large cohort of 427 patients, overall mortality following was 13%; mean carbamazepine ingestion in lethal cases was 23.6 grams.^15^

Most cases of toxicity can be successfully managed with appropriate supportive care including airway protection with endotracheal intubation, treatment of seizures with benzodiazepines, and correction of hypotension with fluid challenges and vasopressors if needed. Hypertonic sodium bicarbonate can be used if evidence of sodium channel blockade is present on the electrocardiography. Gastrointestinal decontamination (e.g., single-dose activated charcoal) is indicated in those patients who present within 1–2 h after ingestion and have no contraindications.^25^

Multiple-dose activated charcoal (MDAC) increases elimination and improves clinical outcome in patients with carbamazepine overdose,^26^ and is recommended for patients with life-threatening ingestions.^27^ However, the use of MDAC can be limited by decreased bowel motility,^23, 28–30^ or concerns over airway protection. Although no antidotes are available to reverse the effects of carbamazepine, isolated case reports have described successful treatment of cardiovascular toxicity with lipid resuscitation therapy.^31,32^ Extracorporeal life support has also been used,^17^ but is technically complicated and not readily available in many hospital settings.

Recommendations from most consulted resources for ECTR in carbamazepine poisoning currently include the presence of life-threatening symptoms unresponsive to conventional treatment,^33–37^ or a contraindication to MDAC.^36^ There are concerns about the effectiveness of ECTR as some articles state that its effect on clearance is not superior to that of MDAC.^37,38^ Therapeutic plasma exchange is mentioned, but evidence is limited and its use is not recommended.^33,34,36^

Oxcarbazepine has a molecular structure and clinical effects that are similar to carbamazepine; however, the scant data^39^ cannot permit reliable extrapolation of the present recommendations to patients with oxcarbazepine poisoning.

## Methodology

Pre-determined methodology incorporated guidelines from The Appraisal of Guidelines for Research and Evaluation (AGREE)^40^ and Grades of Recommendation Assessment, Development and Evaluation (GRADE),^41^ and is described in detail elsewhere.^2^ The primary literature search was conducted on July 12th, 2012 in Medline, Embase, and Cochrane library (Systematic Reviews and CENTRAL).

The search strategy was as follows:

[(carbamazepin*) AND (dialysis OR hemodialysis OR haemodialysis OR hemoperfusion OR haemoperfusion OR plasmapheresis OR plasma exchange OR exchange transfusion OR hemofiltration OR haemofiltration OR hemodiafiltration OR haemodiafiltration OR extracorporeal therapy OR CRRT)]

A manual search in conference proceedings of the EAPCCT and NACCT annual meetings (until 2012), and Google Scholar was performed, as well as the bibliography of each article obtained during the literature search.

A subgroup of EXTRIP completed the literature search, reviewed each article, extracted data, and summarized findings. The level of evidence assigned to each clinical recommendation was determined by the subgroup and epidemiologist (Table 3). Dialyzability was determined based on criteria listed in Table 4. The potential benefit of the procedure was weighed against its cost, availability, alternative treatments, and its related complications. All of this information was submitted to the entire workgroup for consideration, along with structured voting statements based on a pre-determined format.

**Table 3.** Strength of recommendation and level of evidence scaling on clinical outcomes.

Strength of recommendation (consensus-based)	Level of evidence (based on GRADE system)
Level 1 = Strong recommendation = “We recommend…” *The course of action is considered appropriate by the majority of experts with no major dissension. The panel is confident that the desirable effects of adherence to the recommendation outweigh the undesirable effects*Level 2 = Weak recommendation = “We suggest…” *The course of action is considered appropriate by the majority of experts, but some degree of dissension exists among the panel members. The desirable effects of adherence to the recommendation probably outweigh the undesirable effects*Level 3 = Neutral recommendation = “It would be reasonable…” *The course of action could be considered appropriate in the right context*No recommendation *No agreement was reached by the group of experts*	Grade A=High level of evidence *The true effect lies close to our estimate of the effect*Grade B=Moderate level of evidence *The true effect is likely to be close to our estimate of the effect, but there is a possibility that it is substantially different*Grade C=Low level of evidence *The true effect may be substantially different from our estimate of the effect*Grade D=Very low level of evidence *Our estimate of the effect is just a guess, and it is very likely that the true effect is substantially different from our estimate of the effect*

**Table 4.** Criteria of dialyzability.

	Primary criteria	Alternative criteria 1	Alternative criteria 2	Alternative criteria 3
Dialyzability^A^	% Removed^B^	CL_ECTR_/CL_TOT_ (%)^C^	T_1/2 ECTR_/T_1/2_ (%)	Re_ECTR_/Re_TOT_ (%)^C^
**D**, Dialyzable	> 30	> 75	< 25	> 75
**M**, Moderately dialyzable	> 10–30	> 50–75	> 25–50	> 50–75
**S**, Slightly dialyzable	≥ 3–10	≥ 25–50	≥ 50–75	≥ 25–50
**N**, Not dialyzable	< 3	< 25	> 75	< 25

^A^Applicable to all modalities of ECTR, including hemodialysis, hemoperfusion, and hemofiltration.

^B^Corresponds to % removal of ingested dose or total body burden in a 6-hour ECTR period.

^C^Measured during the same period of time.

ECTR = Extracorporeal treatment, CL_ECTR_ = Extracorporeal clearance, CL_TOT_ = Total clearance, RE_ECTR_ = Extracorporeal removal, RE_TOT_ = Total removal, T_1/2 ECTR_ = Half-life with ECTR, T_1/2_ = Half-life without ECTR.

*These criteria should only be applied if measured or calculated (*not reported*) endogenous half-life is > 4h (otherwise, ECTR is not considered clinically relevant). Furthermore, the primary criteria are preferred for poisons having a large Vd (> 5 L/Kg).

Reproduced with permission from Clinical Toxicology

**Fig. 1.**
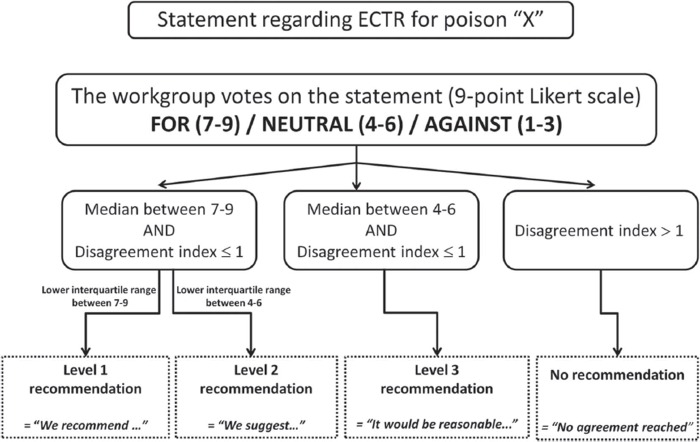
Strength of recommendation algorithm.

The strength of recommendations was evaluated by a two-round modified Delphi method for each proposed voting statement (Fig. 1) and RAND/UCLA Appropriateness Method was used to quantify disagreement between voters.^42^ Anonymous votes with comments were sent to the epidemiologist who then compiled and returned them to each participant. The workgroup met in person to exchange ideas and debate statements. A second vote was later submitted and these results were used in developing the core EXTRIP recommendations. The literature search was updated on October 1st 2014 following the same methodology as described above; the new articles and summarized data were submitted to every participant who then updated their votes.

## Results

**Fig. 2.**
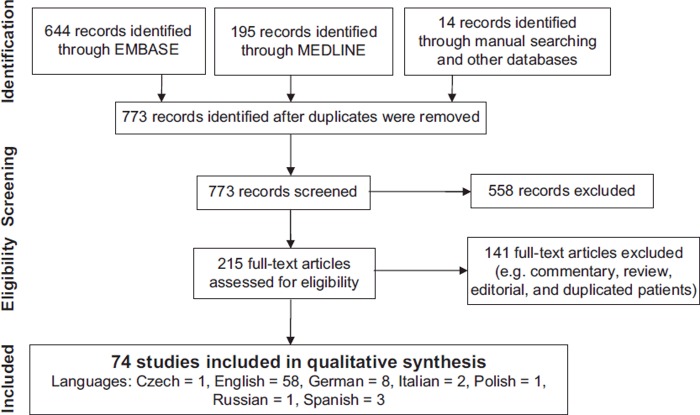
Flow diagram for the literature search (Last performed Oct 1st 2014).

Results of the literature search are presented in Fig. 2. In the final analysis, 74 studies were included: 2 observational studies (83 patients),^43,44^ 65 case reports or case series (71 patients),^8–10,12,16,19,20,23,28–30,45–94,113,118–120^ 4 pharmacokinetic (PK) studies (7 patients),^95–98^ 1 descriptive cohort (12 patients),^99^ and 2 in-vitro studies.^100,101^ No randomized controlled trials were identified.

### Clinical analysis

Two observational studies with clinical outcomes related to ECTR were found in the literature review; the first comparing three different types of ECTR (hemodialysis vs charcoal hemoperfusion vs sorbent hemoperfusion),^44^ and the second comparing low-flux hemodialysis to supportive care.^43^ The former study showed a significantly longer duration of artificial ventilation and worse neurological status at 6 and 12 hours in the group receiving hemodialysis; however, the groups were not comparable at baseline, the hemodialysis cohort being more severely poisoned. Thus, any conclusion on the impact of ECTR on the clinical outcome may have been biased by the presence of confounders. In the latter study, groups were also not comparable prior to the intervention (patients in the ECTR group had a higher carbamazepine concentration and a lower Glasgow Coma Score). Allocation was probably subject to confounding-by-indication (i.e., patients who had more significant neurological findings received hemodialysis). Despite 2 fatalities observed in the HD group, the aforementioned biases again preclude any interpretation on the clinical impact of ECTR. The remaining evidence of a clinical effect of ECTR was composed solely of case reports and case series, with absent control groups, multiple potential confounders, heterogeneous treatments, and definite publication bias. The quality of evidence for all recommendation statements would, therefore, be graded as “very low”.^41^

An aggregate description of case reports is presented in Table 5. The median carbamazepine ingestion and peak concentration were 20.9 grams and 46.2 mg/L, respectively. None of the patients were asymptomatic; all had a varying degree of impairment of consciousness, while several experienced either respiratory depression, hypotension, seizures, conduction abnormalities, or a combination of these. Overall, 6 fatalities were described and appeared consequential to carbamazepine exposure instead of the ECTR itself.^19,20,43,90,99^ Although the evidence may be determined as anecdotal, most of the patients who received ECTR (especially hemoperfusion or hemodialysis) appeared to improve rapidly after initiation of the procedure and had an uneventful outcome, including some patients who reportedly ingested doses greater than 500 mg/kg,^29,30,60,62,64,72,85^ and who had concentrations over 60 mg/L (254 μmol/L).^8,12,16,51,60,66,70,75,79,82,88,120^ Patients receiving less effective treatments, such as continuous renal replacement therapies (CRRT) and peritoneal dialysis, improved less quickly, over days.^56,59,85,118^ Reported complications of ECTRs were almost exclusively associated with hemoperfusion and included hypocalcemia, hypotension, and thrombocytopenia.^10,12,23,28,51,62,70,74,76,77,79,91,92^ Bleeding or a drop in hemoglobin was documented in three cases.^50,70,113^

**Table 5.** Clinical outcomes of the 71 patients described in case reports or case series.

**Patient demographics**	
Mean age (years)	27.2 (range 1.3–58)
Sex (% male)	40.8%
**Poisoning exposure**	
Mean carbamazepine ingestion (grams)	20.9 (0.7–120)
Mean peak carbamazepine concentration (mg/L)	46.2 (range 20–130)
Mean delay between ingestion and admission (hours)	7.8 (range 1–28)
**Clinical symptoms and signs**	
Respiratory depression	39.4%
Decreased consciousness	100%
Seizure (1 or more)	40.8%
Hypotension	18.3%
Dysrhythmias	12.7%
**Other administered**	
MDAC	33.8%
Mechanical ventilation	56.3%
**ECTR**	
Hemodialysis	16.9%
Hemoperfusion	42.2%
Continuous renal replacement therapy	5.6%
Hemoperfusion-Hemodialysis	5.6%
Therapeutic plasma exchange	7.0%
Others	5.6%
More than 1 ECTR	16.9%
**Outcome**	
Sequelae	1.4%
Fatalities	4.2%

MDAC, Multiple-dose activated charcoal; ECTR, Extracorporeal treatment.

*The other fatalities were described in observational cohorts`.

#### Toxicokinetic analysis

Carbamazepine is a small molecule and has a relatively low volume of distribution (V_D_). Protein binding at therapeutic plasma concentration is significant and decreases slightly, if at all, in overdose.^8,9^ Hemoperfusion would, therefore, be the technique most likely to be efficient.^102,103^ Averaged kinetic parameters for all ECTRs (Table 6), kinetic grading of individual patients (Table 7), as well as comparative studies using both hemoperfusion and hemodialysis,^44,64,87^ suggest that hemoperfusion is the most effective technique for removing carbamazepine; both resin and charcoal hemoperfusion have been used successfully with high clearances in this context, although these are sometimes limited by early saturation of the cartridges, an inconsistent finding.^44,53,54,66,72,74,78,79^

**Table 6.** Median clearance for all ECTRs.

Type of ECTRs	N	Median extracorporeal clearance (mL/min)
Albumin dialysis	2	32.9 (range: 18.8–47)
Continuous renal replacement therapy	5	18.5 (range: 16–24)
Hemodialysis	27	59.8 (range: 20–127)
Hemodialysis–hemoperfusion in series	2	97.8 (range: 86.6–109)
Hemoperfusion	28	96.9 (range: 23–173)
Peritoneal dialysis	1	11.7
Exchange transfusion	1	1.5
Therapeutic plasma exchange	1	21.7

*Patients who had more than one ECTR may appear more than once.

N, number of patients

**Table 7.** Kinetic grading for individual patients.

PK/TK grading	TPE	PD	HP	HD	CRRT	Albumin dialysis	HD–HP (in series)	ET
Dialyzable			14	8			1	
Moderately dialyzable			9	20	3	1	2	
Slightly dialyzable	1		3	2	1			
Not dialyzable		1	1	1				1

*Patients who had more than 1 ECTR may appear at more than 1 place.

PK, Pharmacokinetics; TK, Toxicokinetics; TPE, Therapeutic plasma exchange; PD, Peritoneal dialysis; HD, Hemodialysis; HP, Hemoperfusion; CRRT, Continuous renal replacement therapy; ET, Exchange transfusion; ECTR: Extracorporeal treatment.

Nevertheless, carbamazepine appears amenable to removal with other ECTRs, such as hemodialysis and hemofiltration that are not commonly assumed to remove protein-bound molecules.^104^ The median clearances for hemodialysis were inferior to those for hemoperfusion, but the analyzed data included older articles that did not use the modern filters and high blood flows that are standard today. Most recent reports demonstrate comparable clearances with either hemodialysis and hemoperfusion, exceeding 100 mL/min.^16,53,64–66,72,76^ A recent retrospective cohort study suggests a greater initial carbamazepine clearance with charcoal hemoperfusion compared with high-flux hemodialysis, although the effect of hemoperfusion became inferior beyond 3 hours which likely represents saturation of the cartridge.^44^ Hemoperfusion was also performed at much greater cost.^44,99^ Current data cannot determine if a combination of hemoperfusion and hemodialysis in series is preferable to either alone.^49,61,76,79^

The determination of carbamazepine dialyzability is supported by several recent publications,^59,63–65,75,76,97,119^ a number of which collected it directly in dialysate or effluent fluid, which is the preferred method to assess dialyzability.^2,105^ The literature also included a prospective case series of carbamazepine pharmacokinetics in four end-stage renal disease subjects; although the technical characteristics of the ECTR used in the study are outdated (cuprophane filter and low blood flow), clearance during hemodialysis was twice that calculated endogenously.^97^

Based on the criteria defined in Table 4,^2^ most of the cases reviewed for carbamazepine would qualify as either “dialyzable” or “moderately dialyzable.” The workgroup preferred a conservative grading and, therefore, agreed with the following statement: *carbamazepine is moderately dialyzable (level of evidence = B).*

The data on dialyzability of the metabolite carbamazepine-10,11-epoxide are not as abundant as those for carbamazepine. However, its protein binding is less than that of carbamazepine (50%), which suggests at least comparable dialyzability. This hypothesis is supported by a few reports.^8,28,53,60^

### Comparison of ECTR with MDAC

MDAC accelerates the elimination of carbamazepine and is currently supported by the latest position statement jointly published by EAPCCT and AACT.^27^ In the rationale, it is suggested that MDAC provides toxicokinetic advantages similar to those of ECTR.^116–117^ The comparison of carbamazepine toxicokinetics in an individual using different techniques is particularly challenging because its elimination in overdose may follow zero-order or first-order elimination kinetics at different concentrations. Nevertheless, in patients where comparative toxicokinetic estimations (from half-life, clearance, or graphical data plots) were possible, ECTR was superior to MDAC in enhancing elimination of carbamazepine.^9,23,64,66,69,72,76^ In one study in particular, carbamazepine's elimination constant (Ke) was 0.009/h during endogenous metabolism, 0.039/h during MDAC, and 0.059/h during hemodialysis.^9^

## Recommendations (Table 8)

1. General statement: ECTR is suggested in severe carbamazepine poisoning (2D)

**Table 8.** Executive summary of recommendations.

**General statement**
ECTR is suggested in severe carbamazepine poisoning (2D)
**Indications for ECTR**
ECTR is recommended if ANY of the following conditions are present:
○ If multiple seizures refractory to treatment occur (1D)
○ If life-threatening dysrhythmias occur (1D)
ECTR is suggested if ANY of the following conditions are present:
○ If prolonged coma or respiratory depression requiring mechanical ventilation are present or expected (2D)
○ If significant toxicity persists, especially if carbamazepine concentrations rise or remain elevated, despite MDAC and support measures (2D)
**Cessation of ECTR**
Cessation of ECTR is indicated when:
○ Clinical improvement is apparent (1D)
○ Carbamazepine concentration is below 10 mg/L (42 the μ in μmol looks weird/L) (2D)
**Choice of ECTR**
○ Intermittent hemodialysis is the preferred ECTR in carbamazepine poisoning (1D)
○ The following are alternatives if hemodialysis is not available:
• Intermittent hemoperfusion (1D)
• Continuous renal replacement therapy (3D)
**Miscellaneous**
MDAC should be continued during ECTR (1D)

ECTR, Extracorporeal treatment; MDAC, Multiple-dose activated charcoal.

*Rationale*: Carbamazepine is a widely used pharmaceutical and has a narrow therapeutic index. Although rarely fatal, poisoning can cause serious clinical effects and result in a prolonged hospital stay. The data suggesting that rapidly reducing carbamazepine concentration by ECTR may lower morbidity are unavailable, but can be extrapolated from the data on MDAC.^26^ Despite the absence of high-quality evidence, the workgroup considered the following arguments:

- The risk of prolonged coma with mechanical ventilation is not benign.
- Complications associated with ECTR are infrequent and usually mild. There is a theoretical concern of provoking withdrawal seizures in a patient with an underlying seizure disorder.
- There are no life-saving antidotes in carbamazepine poisoning. MDAC may enhance its clearance, but the effect is incomplete and often limited by ileus or concerns of pulmonary aspiration in an unprotected airway.
- ECTR can achieve rapid and substantial removal of carbamazepine.^69,75^
- Although the evidence is anecdotal, patients appear to improve rapidly during ECTR.

For these reasons, the workgroup considered overall that ECTR is worth the risks, costs, and relative uncertainty in patients with severe carbamazepine poisoning, as defined by the conditions below. Most participants (21/28) supported the use of ECTR (voting score, ≥ 7), while the remainder (7/28) had a neutral opinion (voting score, 4–6). While advocating ECTR, the workgroup stated that, in this context, it was unlikely to substantially decrease mortality (as carbamazepine-related fatalities are uncommon) or to avoid irreversible injury (such as it might for blindness in methanol poisoning); instead, ECTR would expectantly be beneficial to reduce short-term morbidity (related to severe hypotension and recurrent seizures) and avoid complications related to prolonged respiratory insufficiency and coma (e.g., ventilator associated pneumonia, pulmonary emboli, and immobilization). Most participants supported the use of ECTR to potentially decrease mechanical ventilation time, intensive care unit (ICU)-related costs, and length of stay in the ICU.^46^ Nevertheless, some participants also considered that active supportive care alone with or without MDAC was sufficient or even preferable to using ECTR in severe carbamazepine poisoning.

2. Indications for ECTR:

ECTR is recommended if ANY of the following conditions are present:

- If intractable seizures occur (1D)
- If life-threatening dysrhythmias occur (1D)

ECTR is suggested if ANY of the following conditions are present:

C. If prolonged coma or respiratory depression requiring mechanical ventilation is present or expected (2D)
D. If significant toxicity persists, especially if carbamazepine concentrations rise or remain elevated, despite MDAC and supportive measures (2D)

*Rationale:* The workgroup proposed that indications for initiation of ECTR in any poisoning should be based on criteria which include exposure (e.g., ingestion, contact, or inhalation), measurement of poison in body fluids, para-clinical tests, and clinical symptoms and signs.

The workgroup agreed that there were too many uncertainties related to the ingested dose to initiate ECTR simply on this information alone. Since serum carbamazepine concentrations are available in the majority of centers, the decision to initiate ECTR should be delayed until confirmation of a toxic exposure becomes possible. Supportive measures, proper gastrointestinal decontamination, and MDAC (as described above) are preferred management for patients presenting after an acute exposure and have no other indication for ECTR. Obviously, if the ingestion history is confirmed and the clinician suspects that major toxicity might ensue (i.e., ingestion > 100 mg/kg and especially > 20 g^15,106^), early communication with a nephrologist and disposition for possible ECTR are warranted.

Monitoring of serum carbamazepine concentrations can confirm exposure and data can be obtained in a time frame relevant enough to guide clinical decisions. Symptoms appear to be more severe at concentrations greater than 40 mg/L (169 μmol/L),^14,24^ although clear correlation with mortality is uncertain.^15^ Nevertheless, the workgroup suggested that isolated elevated carbamazepine concentrations in asymptomatic patients did not warrant ECTR; although several participants stated that some consideration for ECTR should be made at carbamazepine concentrations over 45 mg/L (190 μmol/L), no formal consensus could be obtained at this or any other cutoff. None of the patients identified in our literature review were asymptomatic. Although prophylactic ECTR (i.e., ECTR before the appearance of symptoms) can be considered in poisons where irreversible or life-threatening clinical toxicity can be expected (e.g., paraquat and methanol), the workgroup did not endorse this approach for carbamazepine. The workgroup recognized that children may exhibit more clinical toxicity at an equivalent concentration, perhaps due to a different metabolism and accumulation of the epoxide metabolite,^107–109^ but again the decision to initiate ECTR in this population should be guided on symptoms rather than on an arbitrary serum carbamazepine concentration threshold.

Seizures and dysrhythmias can both follow carbamazepine poisoning and are usually associated with a poorer prognosis.^15^ Obviously, these should be managed with usual supportive therapy as described above. Carbamazepine-induced seizures are possibly induced by accumulation of the epoxide metabolite and are usually singular events when they occur. In the unlikely case that seizures become intractable and unresponsive to conventional therapy, ECTR was strongly recommended by the workgroup. This was also the case for life-threatening dysrhythmias; although the workgroup acknowledged that this was a rare occurrence,^24^ there is some evidence to suggest benefit from rapid carbamazepine removal in this context.^9,12,16^

There was less support for ECTR in carbamazepine-induced coma because this condition was not seen in itself to be as concerning as the above other symptoms. Unlike valproic acid poisoning, where cerebral edema can occur, coma in carbamazepine poisoning is not caused by structural or morphological changes in the CNS. Although not necessarily associated with a poor outcome,^15,24^ coma can be particularly prolonged in massive ingestions and might necessitate protracted mechanical ventilation, in which case there was more agreement to initiate ECTR. Although difficult to estimate clinically, a protracted course can be anticipated in patients with large ingestions, delayed-release preparations, extremely high concentrations, and those who are naïve to carbamazepine (absence of auto-induction in patients on chronic therapy).

Lesser symptoms such as ataxia, ileus, confusion, nystagmus, and mild cardiac conduction defects are associated with a favorable outcome^15^ and were not considered sufficiently severe to justify ECTR.

Increasing carbamazepine concentrations are usually reflective of prolonged absorption which can be managed by MDAC. The inability to administer MDAC was not considered by itself as an indication for ECTR but would reduce its decision threshold for initiation in toxic patients. However, in cases where MDAC is contraindicated, unavailable, could not be adequately performed or unable to reduce carbamazepine concentrations, and with evidence of clinical toxicity, ECTR was recommended by the workgroup. This was also the case in patients who fail supportive therapy.

3. Cessation of ECTR: ECTR is indicated until sustained clinical improvement is apparent (1D) or carbamazepine serum concentration is below 10 mg/L (42 μmol/L) (2D)

*Rationale:* One of the intents of beginning ECTR in patients with carbamazepine poisoning is to reduce complications associated with prolonged coma and hypoventilation. It is, therefore, reasonable to pursue ECTR until clinical improvement becomes apparent. This improvement should be sustained for a period of time long enough to account for any ongoing absorption. Although a high carbamazepine concentration is not by itself an indication of initiating ECTR, the workgroup agreed that below 10 mg/L (42 μmol/L), most toxic symptoms would be expected to resolve.

Clinicians should continue to monitor carbamazepine concentrations regularly after ECTR, as concentrations may rebound. This was present in one-third of patients reviewed from our cohort and is particularly concerning if caused by ongoing absorption,^76^ as improvement of ileus will in turn augment carbamazepine absorption. If concentrations rebound to threatening levels or if there is recurrence of toxic symptoms, another ECTR session may be indicated. For this reason, the central venous catheter should only be removed once the clinician is assured that ECTR is no longer needed.

4. Choice of ECTR: Intermittent hemodialysis is the preferred modality for ECTR in carbamazepine poisoning (1D). Intermittent hemoperfusion (1D) and continuous renal replacement modalities (3D) are alternatives if intermittent hemodialysis is not available.

*Rationale*: The workgroup agreed that hemodialysis is the preferred modality of ECTR in carbamazepine poisoning. Although historical preference was given to intermittent hemoperfusion, because of its theoretical advantage of clearing protein-bound poisons such as carbamazepine, there are several arguments that now favor the use of intermittent hemodialysis:

- Recent data suggest that hemodialysis is almost as effective as hemoperfusion^9,64,87^ due to improved clearances provided by newer high-flux and high-efficiency synthetic membranes compared with those provided by older less efficient cuprophane dialyzers. Present-day catheters can also achieve blood flows during hemodialysis up to 400 mL/min, while blood flow for several hemoperfusion cartridges remains limited to 300–350 mL/min^68^ because of the risk of hemolysis.^110^
- Intermittent hemodialysis is the favored treatment for maintenance dialysis in patients with end-stage kidney disease (ESRD) and acute kidney injury (AKI) worldwide; therefore, this is the most available modality. Meanwhile, hemoperfusion cartridges are of limited availability in many parts of the world, as is the accessibility to online hemofiltration. Therefore, the travel distance to a hemodialysis center for a poisoned patient would likely be minimized.
- More physicians and nurses are experienced with hemodialysis, with lesser risks of delay and uncertainty.
- The complication rate with hemodialysis appears favorable in comparison to hemoperfusion.^111^ At least one third of the patients evaluated in the present cohort experienced thrombocytopenia during hemoperfusion.
- The cost of hemodialysis favors it over hemoperfusion. This is largely explained by the cost of monitoring and treating complications as well as the lower cost of dialysis filters versus charcoal cartridges, which need to be replaced regularly because of saturation of its adsorptive capacity.^44^

Nevertheless, in the rare case where hemoperfusion, but not hemodialysis is available, it would be an acceptable alternative, if cartridges are available and if physicians and nursing personnel are comfortable with this technique. Similarly, it is anticipated that carbamazepine would be removed by intermittent convection-based techniques (online HF).^62^

Continuous techniques offer markedly lower clearances and removal rates compared with intermittent techniques^60,76,85,113,119^ and, as such, are considered to be inferior modalities by EXTRIP and only advocated if hemodialysis is not available (due to technical or staffing reasons in critical care settings). Continuous techniques are usually better tolerated hemodynamically than intermittent techniques, although this is mostly true in cases where net fluid removal is necessary, which would be an unusual requirement in carbamazepine poisoning. Peritoneal dialysis,^59^ exchange transfusion,^95^ and plasma exchange^63^ do not offer comparable results to hemodialysis or hemoperfusion, as expected,^112^ and are not currently recommended. Limited data for albumin-enhanced techniques are available,^46,73,113,119^ but these procedures are extremely costly and have not shown superiority to either hemoperfusion or hemodialysis; in one study, the addition of 20% albumin in the dialysate increased clearance by 24%.^119^

Whatever the technique is used, operating ECTR characteristics should be optimized to maximize removal, that is, high blood and dialysate flow rates,^104,114,119^ high surface area filters, and longer duration of ECTR.^104^

5. Miscellaneous: MDAC therapy should be continued during ECTR (1D)

*Rationale:* MDAC may enhance elimination of carbamazepine, may reduce toxicity in carbamazepine-poisoned patients,^26^ and is currently recommended for this indication by various toxicology societies.^27^ The workgroup supported MDAC during ECTR in patients who did not present contraindications to its use. Its effect on elimination would likely be additive to ECTR and should be used whenever the airway is protected, either by the patient's own reflexes or an endotracheal tube.^9,64,72^

## Conclusion

Here, the EXTRIP workgroup presents its recommendations for ECTRs in carbamazepine poisoning. The evidence suggests that elimination enhancement with intermittent hemodialysis or hemoperfusion is superior to MDAC. In the large majority of patients with toxic carbamazepine exposures, supportive management appears sufficient. However, in severe cases, the group supported the use of ECTR on the basis that advantages superseded costs and risks associated with the procedures, despite an absence of robust clinical studies.

## Notice of Correction

Since this article was posted online on 30 Oct 2014 there have been revisions to the figures, text and tables.
